# A genome-wide screen in *Pseudomonas aeruginosa* identifies genes impacting production of the hemolytic phospholipase C/sphingomyelinase, PlcH

**DOI:** 10.1128/jb.00231-26

**Published:** 2026-07-27

**Authors:** Kristin Schutz, Olivia F. Evans, Jacob R. Mackinder, Pauline DiGianivittorio, Aditya Patwardhan, Matthew J. Wargo

**Affiliations:** 1 Department of Microbiology and Molecular Genetics, Larner College of Medicine, University of Vermont 2092, Burlington, Vermont, USA; 2 Cellular, Molecular, and Biomedical Sciences Graduate Program, University of Vermont 2092, Burlington, Vermont, USA; Dartmouth College Geisel School of Medicine, Hanover, New Hampshire, USA

**Keywords:** sphingosine, pathogenesis, phospholipase C

## Abstract

The secreted phospholipase C/sphingomyelinase, PlcH, is the heat-labile hemolysin of *Pseudomonas aeruginosa* and one of its important secreted virulence factors. While there are known and suspected genes that impact PlcH production in *P. aeruginosa*, we sought to identify additional genes by screening the PA14 transposon mutant library to measure extracellular PlcH enzyme activity induced by choline. The library as a whole had a log2-normal distribution of nitrophenylphosphorylcholine hydrolysis activity, with the genes of interest being those in long tails well outside of the distribution. These outlier genes included nearly all of those known to be important for PlcH production in response to choline, including those required for choline metabolism, glycine betaine sensing, and secretion through the outer membrane. Interestingly, higher PlcH production was also seen in mutants of the protease-associated genes *lon*, *mucD*, and *clpA*, as well as other genes. Additionally, we identified genes impacting baseline levels of PlcH production, which include genes in the dimethylglycine metabolism locus involved in choline metabolism. The high hit rate of known and suspected genes supports the power of this screen. Additionally, our verification of these genes by clean deletion in strain PA14 confirms the broad importance of these systems across *P. aeruginosa*, as previous work was confined to strain PAO1. There were many genes identified in this screen that were not individually examined, and the complete screen results reported here should allow others to identify the intersection of their genes of interest with PlcH production.

**IMPORTANCE:**

*Pseudomonas aeruginosa* is an important opportunistic pathogen that employs multiple independent virulence factors to cause infection, one of which is the hemolytic phospholipase C/sphingomyelinase PlcH. Using a whole-genome screen, we identified both known and previously unknown genes contributing to *P. aeruginosa* PlcH production. Our findings provide insight into the integration of various cellular processes with PlcH production and identify potential genes that may impact the PlcH expression heterogeneity seen in *P. aeruginosa* clinical isolates.

## INTRODUCTION

*Pseudomonas aeruginosa* is a common opportunistic pathogen, particularly in healthcare settings, that causes infections in a variety of body sites including the skin, eyes, lungs, and bloodstream. Morbidity and mortality due to *P. aeruginosa* infections are driven in part by a collection of virulence factors secreted either directly into host cells by the type 3 and type 6 secretion systems or into the extracellular milieu via other secretion systems, where they can directly impact host molecules and cells (1–3). One of these extracellular secreted virulence factors is the heat-labile hemolysin (4), PlcH, which is a secreted phospholipase C/sphingomyelinase (5). PlcH expression is retained in all clinical strains tested (6–9), and higher PlcH production by strains has been negatively correlated with patient outcomes and azithromycin efficacy in cystic fibrosis (9, 10). Mutants in *plcH* are less virulent in infection models (11–16), and PlcH directly drives cytotoxicity, inflammation, and pulmonary surfactant damage (11, 17–21).

**Fig 1:**
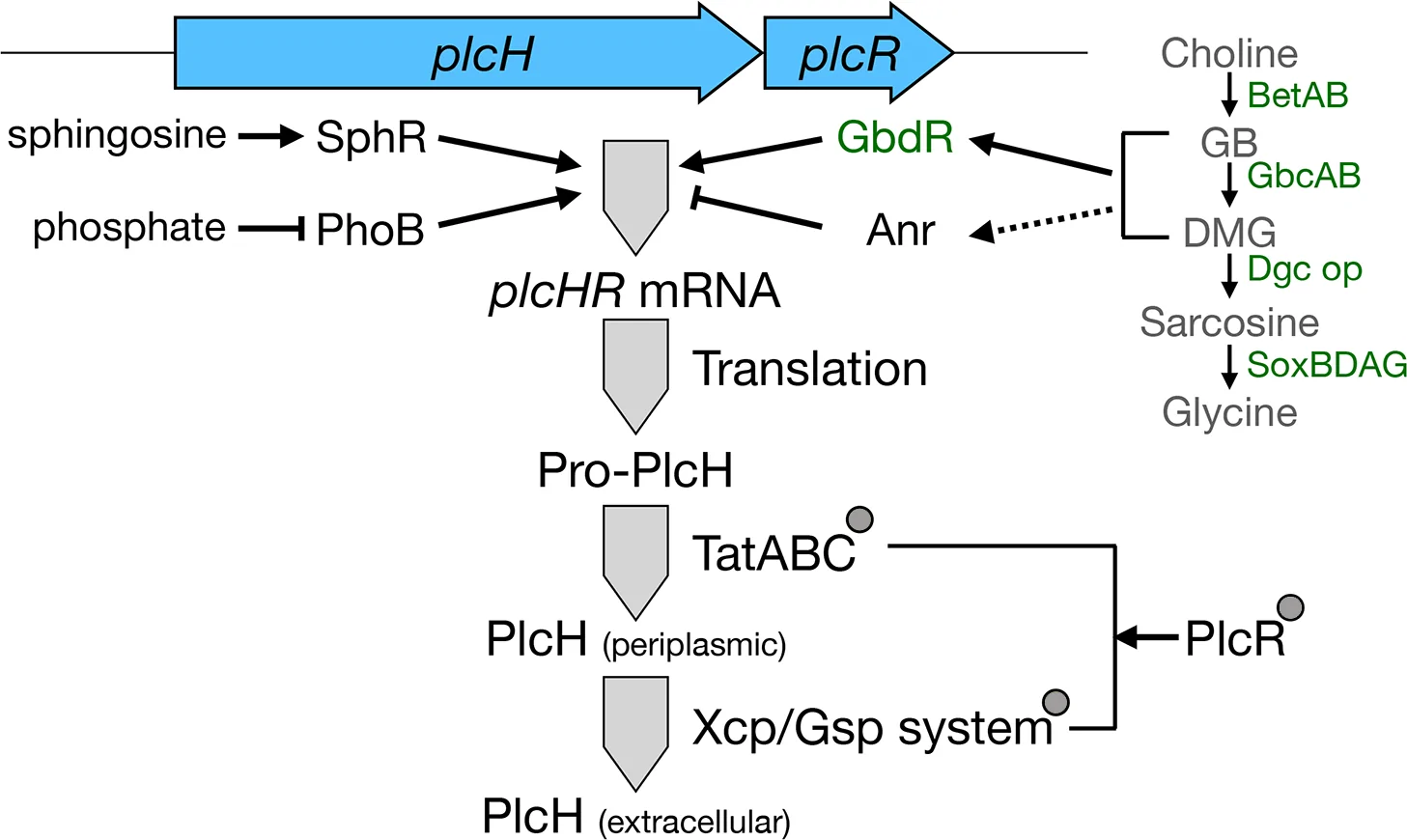
Proteins known to contribute to PlcH production. This schematic illustrates the proteins and small molecules contributing at the various stages of PlcH production, starting with transcription at the top and finishing with mature, extracellular PlcH at the bottom. Text in green are the proteins involved in choline catabolism and the GB and DMG induction of *plcHR* transcription. The gray circles to the right of the text indicate that these were previously demonstrated only in a single *P. aeruginosa* strain—PAO1 only for these three systems.

The production of PlcH is under regulation at a number of steps, with known direct contributors illustrated in Fig. 1. PlcH is secreted from the cytosol to the periplasm via the twin-arginine translocase (TAT) system (22) and secreted from the periplasm to the extracellular milieu via the general secretion pore (Gsp, also called Xcp in *P. aeruginosa*) (23, 24). PlcH secretion is aided by the PlcR1 and PlcR2 chaperones, both encoded by the *plcR* gene in the *plcHR* operon (25). Transcription of *plcH* can be induced by phosphate starvation via PhoB (26, 27), glycine betaine (GB) and dimethylglycine (DMG) via GbdR (28), and sphingosine via SphR (29). Choline can induce *plcH* transcription in a *gbdR*-dependent manner, but only once it has been metabolized to GB, as mutations in the choline oxidase, *betA*, prevent *plcH* induction by choline (30). Induction of *plcH* by sphingosine via SphR occurs due to transcriptional readthrough from the upstream *cerN* promoter (29). Anr also regulates *plcH* transcription, and this regulation is impacted by GB metabolism (31, 32). Transcriptional induction of *plcHR* by GB can be regulated by catabolite repression control (33), likely due to catabolite repression of *gbdR* transcription (34), though this has not been definitively tested, and it is important to note that many clinical strains have mutations in or quickly lose catabolite repression during infection (35). There is a Vfr binding site in the *cerN-plcH* intergenic region, though no function due to this site has been previously shown, and Vfr was previously ruled out as the mediator of *plcH* catabolite repression (33). The H-NS family proteins MvaU and MvaT are known to regulate expression from many loci in *P. aeruginosa*, directly or indirectly, including *cerN* and *plcH* (36). PhrS has also been shown to negatively regulate *plcH* expression, though likely indirectly (37).

Given these known and suspected genes impacting PlcH activity and the potential that other genes also contribute, we set out to identify transposon mutants with altered PlcH production by screening the *P. aeruginosa* PA14 transposon mutant library for PlcH activity by measuring hydrolysis of the colorimetric substrate nitrophenylphosphorylcholine (NPPC). Here, we report on the outcomes of that screen, further characterization of selected genes, and highlight some previously unsuspected genes that impact PlcH production in *P. aeruginosa*.

## RESULTS

### Screen of the PA14 TnM library for altered choline-dependent induction of PlcH enzyme activity

**Fig 2:**
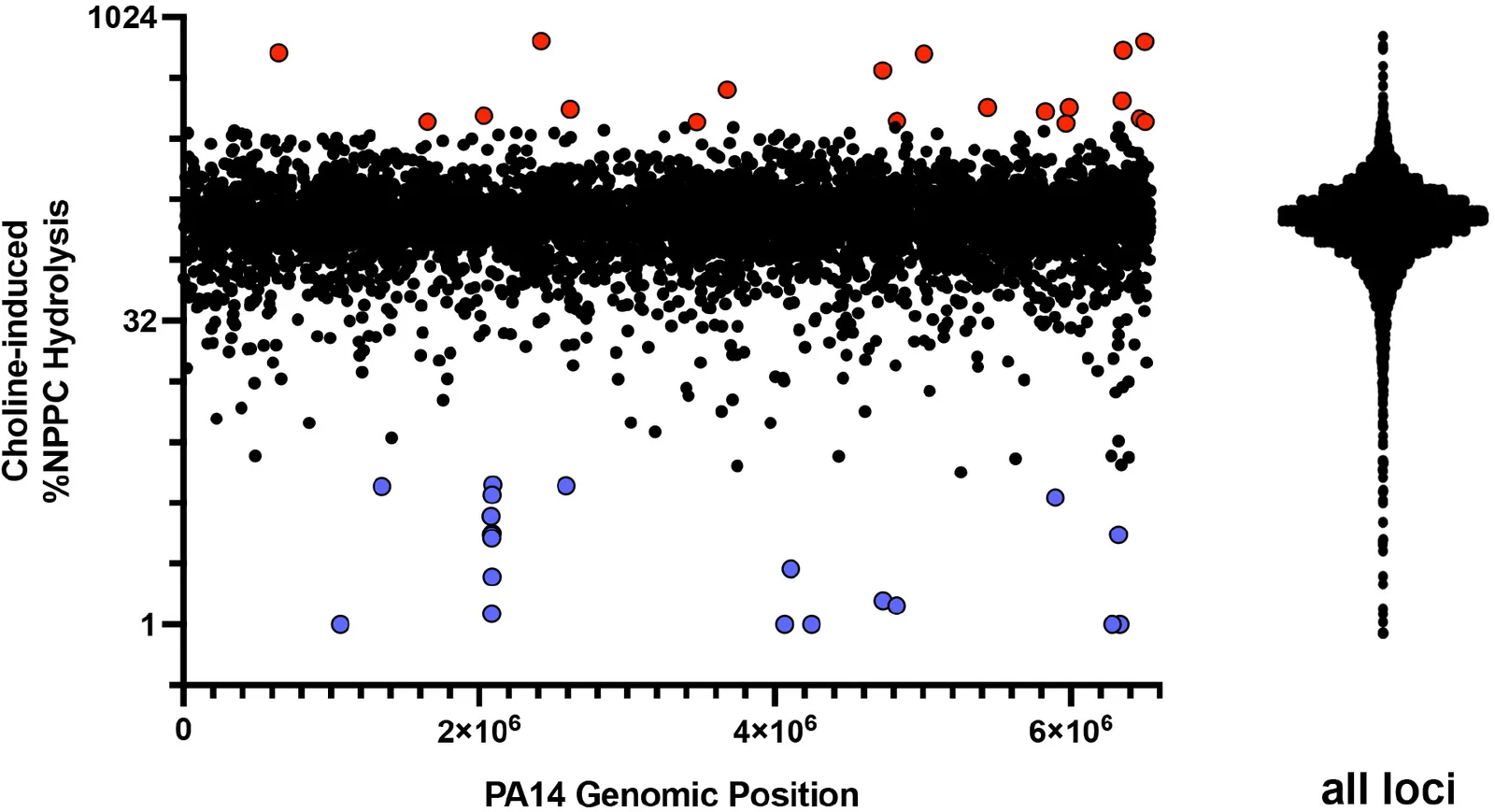
Screen of the *P. aeruginosa* PA14 transposon mutant library for choline-induced NPPC hydrolysis activity. The NPPC hydrolysis activities for each transposon mutant are plotted on a log2 scale after normalization to the within-plate average. On the left, expression is mapped versus the genomic position of each transposon insertion. Red points are those with the highest NPPC hydrolysis activity, while blue points are those showing the lowest NPPC hydrolysis activity, both classes of which are detailed in Table 1. To the right is the single-column scatter plot of the same data showing the normal distribution of the transposon insertant activities.

We conducted NPPC hydrolysis assays on the entire PA14 mutant library (38), as described in detail in the Materials and Methods, to identify mutants with altered extracellular PlcH enzyme activity. The PA14 library is a mostly non-redundant library of non-essential genes in PA14, containing 5,459 single-insertion transposon mutants in 4,596 genes (1,366 genes not represented) (38). The NPPC assay is a sensitive proxy for PlcH activity, particularly using choline as the only inducing signal, as other NPPC-hydrolyzing enzymes (particularly PlcN) are not induced under these conditions (27, 30, 33, 39). The within-plate normalized choline-induced NPPC hydrolysis activity shows a normal distribution when log2 transformed, with a log2 mean of 6.57 (95.2% linear), a median of 6.66 (101.1% linear), and a standard deviation of 0.73 (Fig. 2). The transposon mutants with the highest and lowest NPPC hydrolysis activities are listed with additional details in Table 1. Complete data for this screen are available in Table S1, which includes Z-scores. Insertants that were determined to be auxotrophs or determined to be cross-contaminants by sequencing were removed from Fig. 2 and Table 1. The two instances of cross-contamination are noted in the text in Table S1, while auxotrophs have “NA” listed in the activity column—described more in the Materials and Methods section.

**TABLE 1:** List of transposon mutants with the highest and lowest levels of choline-induced NPPC hydrolysis activity*^a^*^,*b*^

%NPPC hydrolysis	PA14 locus	PAO1 ortholog	PA14 NR plate:well	Tn insertion position bp (full gene length bp)	“Active” gene name	“Active” gene description
782.1	PA14_27950	PA2797	11_2:E2	268 (483)	hyp	Putative anti-anti-sigma factor
775.4	PA14_72990	PA5533	6_2:G10	261 (369)	hyp	Conserved hypothetical protein
702.7	PA14_71280	PA5399	5_2:H9	1,492 (1,962)	***dgcB***	Putative ferredoxin
683.2	PA14_07500	PA0575	8_3:B8	335 (3,738)	*rmcA*	Conserved hypothetical protein
673.6	PA14_56070	PA4315	6_3:B7	254 (375)	***mvaT***	Transcriptional regulator MvaT, P16 subunit
559.2	PA14_53340	PA0845	10_3:F1	1,916 (2,013)	***cerN***	Neutral ceramidase
448.4	PA14_41220	PA1803	4_1:A9	409 (2,397)	*lon*	Lon protease
395.1	PA14_71240	PA5396	9_2:F12	122 (978)	***PA5396***	Putative dipeptidase
366.5	PA14_60950	PA4606	7_4:H11	10 (2,067)	hyp	Carbon starvation protein
365.1	PA14_67090	PA5078	3_3:F7	158 (1,578)	*mdoG*	Periplasmic glucan biosynthesis protein, MdoG
358.8	PA14_30230	PA2620	8_4:C11	180 (2,277)	*clpA*	ATP-dependent clp protease, ATP-binding subunit
349.4	PA14_65410	PA4951	14_3:C1	22 (543)	*orn*	Oligoribonuclease
332.9	PA14_23400		9_3:H7	1,330 (1,350)	*ORF_8*	Predicted O-antigen synthesis pathway
322.2	PA14_72560	PA5499	10_3:C3	4 (504)	*zur/np20*	Transcriptional regulator zur/np20
312.7	PA14_54390	PA0766	6_3:F7	35 (1,425)	*mucD*	Serine protease MucD precursor
311.1	PA14_73020	PA5536	5_3:F3	40 (405)	*dksA2*	Putative C4-type zinc finger, DksA/TraR family
310.8	PA14_19110	PA3478	14_4:H8	805 (1,281)	*rhlB*	Rhamnosyltransferase chain B
310.3	PA14_38950	PA1977	3_4:H7	478 (864)	hyp	Putative transmembrane protein, EmrE family
305.0	PA14_66820	PA5056	12_1:H3	269 (1,680)	*phaC1*	Poly(3-hydroxyalkanoic acid) synthase 1
						
4.9	PA14_24100	PA3095	10_1:B4	142 (525)	***xcpZ***	General secretion pathway protein M
4.9	PA14_29880	PA2647	5_3:A12	1,196 (1,848)	*nuoL*	NADH dehydrogenase I chain L
4.8	PA14_15750		10_1:G6	87 (979)	hyp	Conserved hypothetical protein
4.4	PA14_24020	PA3101	6_2:D7	190 (447)	***xcpT***	General secretion pathway protein G
4.2	PA14_66160	PA5004	5_1:G10	79 (1,137)	*wapH*	Putative glycosyl transferase
3.4	PA14_23970	PA3105	10_4:E10	1,847 (1,977)	***xcpQ***	General secretion pathway protein D
2.8	PA14_24060	PA3098	9_1:E9	226 (714)	***xcpW***	General secretion pathway protein J
2.8	PA14_70940	PA5372	9_3:B6	52 (1,686)	***betA***	Choline dehydrogenase
2.8	PA14_23970	PA3105	2_2:H7	50 (1,977)	***xcpQ***	General secretion pathway protein D
2.7	PA14_23990	PA3103	15_3:D11	571 (1,509)	***xcpR***	General secretion pathway protein E
1.9	PA14_46200	PA1411	2_4:A12	6 (912)	hyp	Putative membrane protein, EmrE family
1.7	PA14_24020	PA3101	2_1:F12	377 (447)	***xcpT***	General secretion pathway protein G
1.3	PA14_53360	PA0844	12_4:C7	287 (2,193)	***plcH***	Hemolytic phospholipase C precursor
1.2	PA14_54370	PA0767	8_3:A11	616 (1,800)	*lepA*	GTP-binding protein LepA
1.1	PA14_23990	PA3103	12_3:C4	820 (1,509)	***xcpR***	General secretion pathway protein E
1.0	PA14_12300	PA3983	5_4:A7	92 (840)	*corC*	Putative Mg^2+^ and Co^2+^ transporter CorC
1.0	PA14_45710	PA1450	15_2:A7	811 (1,260)	hyp	Conserved hypothetical protein
1.0	PA14_71070	PA5380	1_3:G2	160 (1,104)	***gbdR***	Putative transcriptional regulator, AraC family
1.0	PA14_70480	PA5339	6_2:F12	88 (381)	hyp	Putative endoribonuclease L-PSP
1.0	PA14_47730	PA1275	6_3:F10	520 (939)	*cobD*	Cobalamin biosynthetic protein CobD

^*a*^ %NPPC hydrolysis normalized within plate; percentages below 1% converted to 1% for log2 transformation to graph in Fig. 2.

^*b*^ Bolded gene names are those previously known or predicted to impact PlcH production.

### Transposon insertions in known PlcH secretion and export systems, along with choline metabolism genes, validate the screen

Transposon insertion into genes previously known to impact PlcH expression and activity provided internal validation for the screen. For the data in this paragraph, we are referring to Fig. 2, Table 1, and Table S1. The transposon insertion into *plcH* shows very low NPPC hydrolysis activity, supporting our NPPC hydrolysis assay as predominately measuring PlcH enzyme activity under these conditions. This conclusion is also supported by the insertion mutant in *gbdR*, which is required for choline-dependent induction of *plcH* transcription (28). Transposon insertion in *betA* and, to a lesser extent, *betB*, which encode the choline oxidase and betaine aldehyde dehydrogenase, respectively, results in reduced PlcH induction by choline, as expected (30). We previously showed that chemical inhibition of the dimethylglycine catabolism enzyme, DgcA, or deletion of the *dgcA* gene in strain PAO1 led to hyperinduction of *plcH* transcription in the presence of choline (40), and conversely, that overexpression of the GB catabolic genes *gbcA* and *gbcB* led to reduced PlcH expression (41). Correspondingly, transposon insertions in the dimethylglycine catabolic operon (*dgcB* and *PA5396*) show the expected hyperinduction phenotype. At this time point in PA14, transposon insertions into *gbcA* and *gbcB* did not show hyperinduction of PlcH.

Transposon insertions into nearly every gene in the *xcp* locus were defective for extracellular PlcH activity, supporting previous findings that PlcH is exported from the periplasm by the Xcp/Gsp system (23). The *xcp* transposon mutants also demonstrate that NPPC is not significantly transported into the periplasm in PA14, where PlcH remains active (23, 24). One system required for PlcH expression that was not identified in this screen was the TAT secretion system encoded by *tatABC* (42). Though they are not essential (42, 43), there are no insertants in *tatA* or *tatB* in the PA14 transposon library, and the *tatC* insertion in our library copy did not show any of the known TAT phenotypes (42). We determined that our *tatC* well was contaminated by a transposon mutant from an adjacent well, and no *tatC* insertion was detectable by PCR from that well.

**Fig 3:**
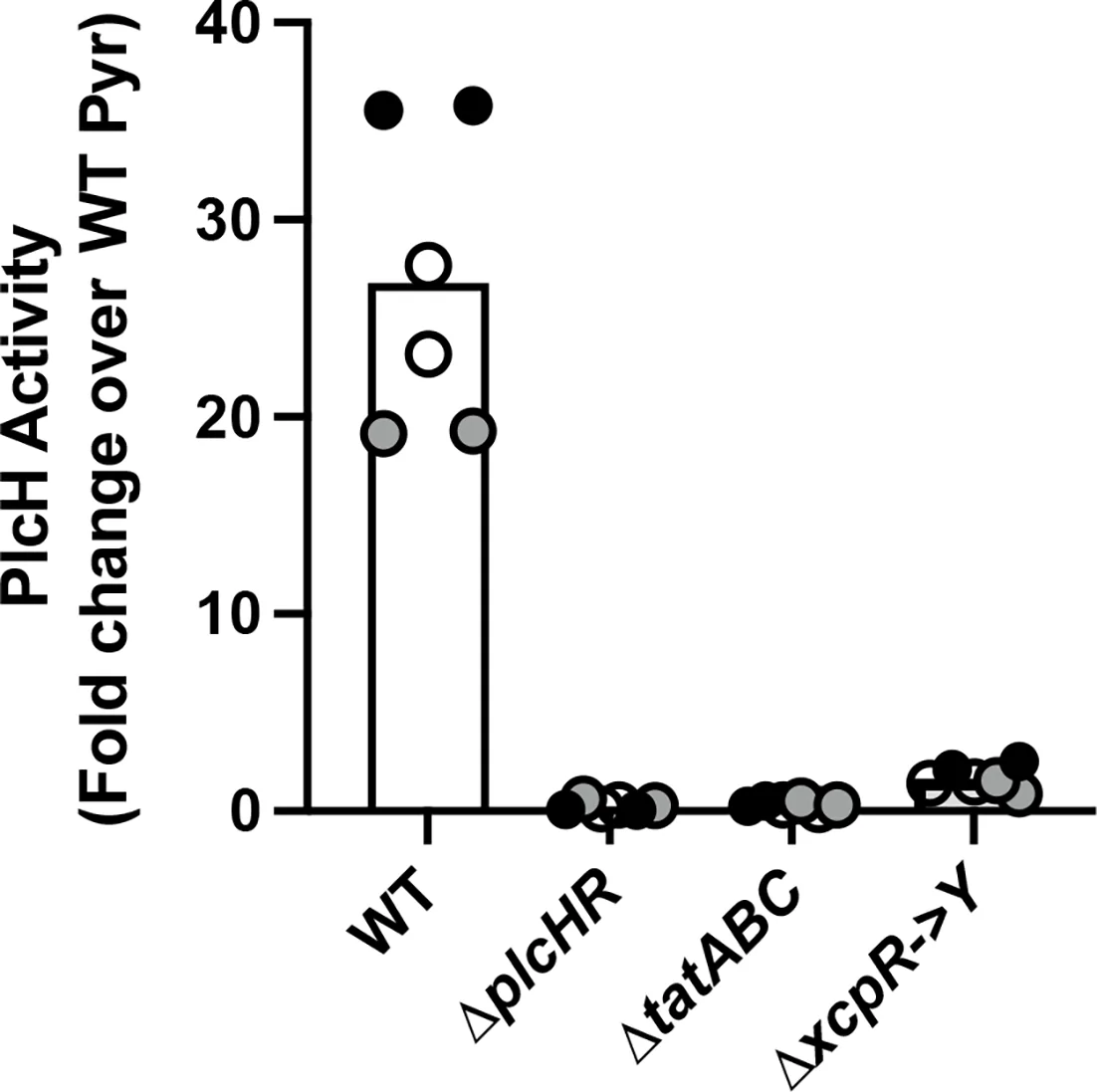
Secreted PlcH activity in mutants of the TAT secretion system and Xcp/Gsp system. The fold change in PlcH activity is plotted as measured by NPPC hydrolysis after choline induction over the hydrolysis in the non-inducing pyruvate control condition. The ∆*plcHR*, ∆*tatABC*, and ∆*xcpRSTUVWXY* (∆*xcpR→Y*) strains are all statistically different than WT with *P* < 0.0001, determined by ANOVA with Dunnett’s post-test using WT as the comparator. All data points are shown and are colored by experiment, with white circles for all replicates from experiment #1, gray from experiment #2, and black from experiment #3, with the overall means represented by the bars. Only the means from each experiment are used in the statistical analyses for these panels (i.e., *n* = 3 per condition).

To verify the role of TAT and Xcp in PA14 PlcH secretion, as most prior work was conducted in *P. aeruginosa* strain PAO1 and the above data are from transposon mutants, we generated both *tatABC* and *xcpRSTUVWXY* deletion mutants in *P. aeruginosa* PA14 and measured extracellular NPPC hydrolysis activity compared to wild-type (Fig. 3), which showed that both systems are required for functional extracellular PlcH, in concordance with results in PAO1 (23, 24, 42).

Altogether, the data from mutants in the choline pathway, the *xcp* locus, and the TAT system support their roles in PlcH activity and provide validation for the screen.

### Secondary screen for transposon mutants not further explored in this study

**Fig 4:**
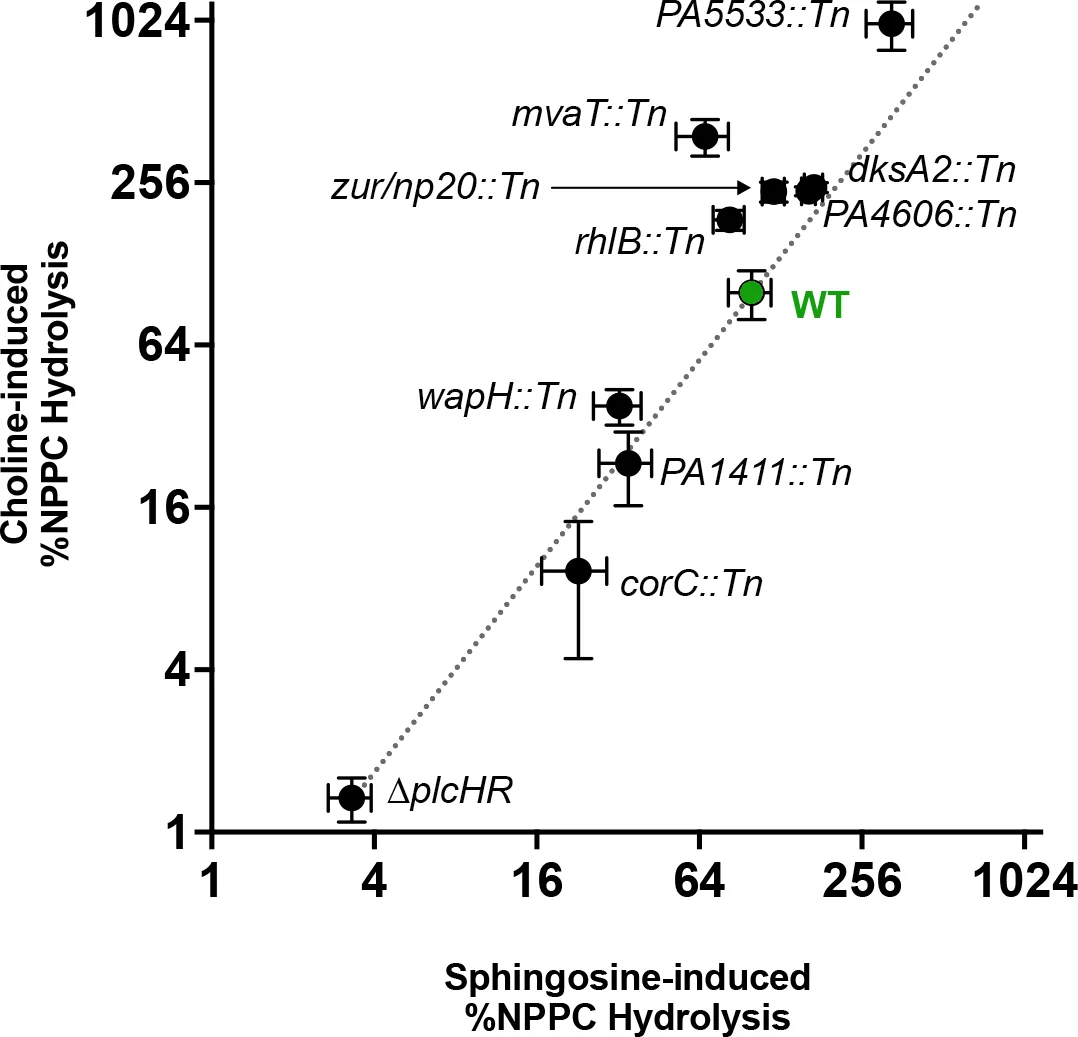
Secondary screen of selected transposon mutants for relative PlcH production. The NPPC hydrolysis activity induced by choline (*y*-axis) or sphingosine (*x*-axis) of a selection of transposon mutants was normalized to the levels of PA14 WT (in green). Error bars represent the standard deviation of three independent biological replicates. The dotted line indicates the roughly log-linear relationship for most mutants, with the line originating at ∆*plcHR* and passing through the center of the PA14 WT.

We selected a subset of transposon mutants that were, for the most part, not further examined in this study and assessed PlcH activity induced by choline compared to induction by sphingosine. Since these two inducing conditions are dependent upon different regulators (28, 29), they allow orthologous assessment of whether impacts are likely transcriptional or likely post-transcriptional. As seen in Fig. 4, there is a rough log-linear relationship between induction in each condition for most of these mutants, suggesting that their impact on PlcH expression is independent of the induction method (i.e., likely post-transcriptional). The two exceptions to this pattern to note are *mvaT::Tn* and *rhlB::Tn*, both of which show higher PlcH induction in choline than WT, but similar or lower induction in response to sphingosine. After confirmation of these phenotypes using the secondary screen, we explored a number of other genes identified in Table 1 throughout the remainder of the Results section.

### Transposon insertions that lead to altered baseline (pyruvate) NPPC hydrolysis activity

Most transposon mutants, including many of those with altered PlcH activity in response to choline, maintained normal NPPC hydrolysis activity in the uninduced condition (pyruvate, also described as baseline); however, a few showed marked induction at baseline (Table 2; Table S1). The top transposon mutant altered for baseline PlcH production was an insertion into *cerN*. That particular insertion is at the extreme 3′ end of the *cerN* gene, and our prediction is that the transposon internal promoter is constitutively driving baseline transcription of *plcH*, which lies immediately downstream of *cerN*. Deletion of *cerN* led to increased choline-dependent PlcH production, but that effect was not complemented in *trans,* and the deletion did not have increased basal induction or increased induction in response to sphingosine (Fig. S1). Thus, the *cerN::Tn* baseline expression phenotype is likely dependent upon the position and presence of the transposon. However, there is a specific effect of the *cerN* deletion on choline-induced PlcH expression, but not sphingosine induction, suggesting some impact on GbdR function at the *plcH* promoter upon *cerN* deletion.

**TABLE 2:** Transposon mutants with more than fivefold higher NPPC hydrolysis activity than WT in baseline (pyruvate, uninduced) conditions

Baseline (Pyr) %NPPC hydrolysis activity	Choline-induced %NPPC hydrolysis activity	PA14 locus	PAO1 ortholog	PA14 NR plate:well	Gene name	Gene description
30,890.3	559.2	PA14_53340	PA0845	10_3:F1	*cerN*	Alkaline ceramidase
6,242.3	702.7	PA14_71280	PA5399	05_2:H9	*dgcB*	Putative ferredoxin
1,043.7	775.4	PA14_72990	PA5533	06_2:G10		Conserved hypothetical protein
834.7	262.2	PA14_70850	PA5368	05_2:H12	*pstC*	Phosphate ABC transporter, permease protein
711.6	118.1	PA14_21090	PA3320	03_1:D10		Conserved hypothetical protein
708.2	142.4	PA14_71460	PA5415	01_2:E9	*glyA1*	Serine hydroxymethyltransferase
639.0	191.5	PA14_55820	PA4297	08_3:H7		Putative membrane protein
628.0	349.4	PA14_65410	PA4951	14_3:C1	*orn*	Oligoribonuclease
610.6	683.2	PA14_07500	PA0575	08_3:B8	*rmcA*	Conserved hypothetical protein
607.6	189.3	PA14_58470	PA4505	03_4:C8	*dppD*	Putative dipeptide ABC transporter
587.1	322.2	PA14_72560	PA5499	10_3:C3	*zur/np20*	Transcriptional regulator zur/np20
583.4	153.2	PA14_58600	PA4516	03_1:C3		Conserved hypothetical protein
554.8	144.2	PA14_43630	PA1615	03_1:B7		Putative lipase
521.7	97.9	PA14_00730	PA0061	04_4:A12		Conserved hypothetical protein
504.0	234.0	PA14_55060	PA0987	06_4:B1		Conserved hypothetical protein

Transposon insertion into *PA5533* led to increased baseline and induced levels of PlcH activity in response to both choline and sphingosine (Tables 1 and 2; Fig. 4), but deletion of this gene did not alter PlcH activity (Fig. S2). The impact of transposon insertion into *PA5533* may be due to its proximity to the zinc regulator, *zur*, insertion into which showed a PlcH production phenotype (Table 1; Fig. 4; Table 2; Table S1). The increased baseline phenotype of the *pstC* insertant is expected based on the constitutive activation of PhoB in this mutant, which is an activator of *plcH* transcription (27, 44). The increased baseline phenotype for the *dgcB* and *glyA1* insertants will be covered later in the Results section. We have not followed up on the other genes in Table 2 at this time.

### Mutations in some protease-related genes enhance PlcH expression

**Fig 5:**
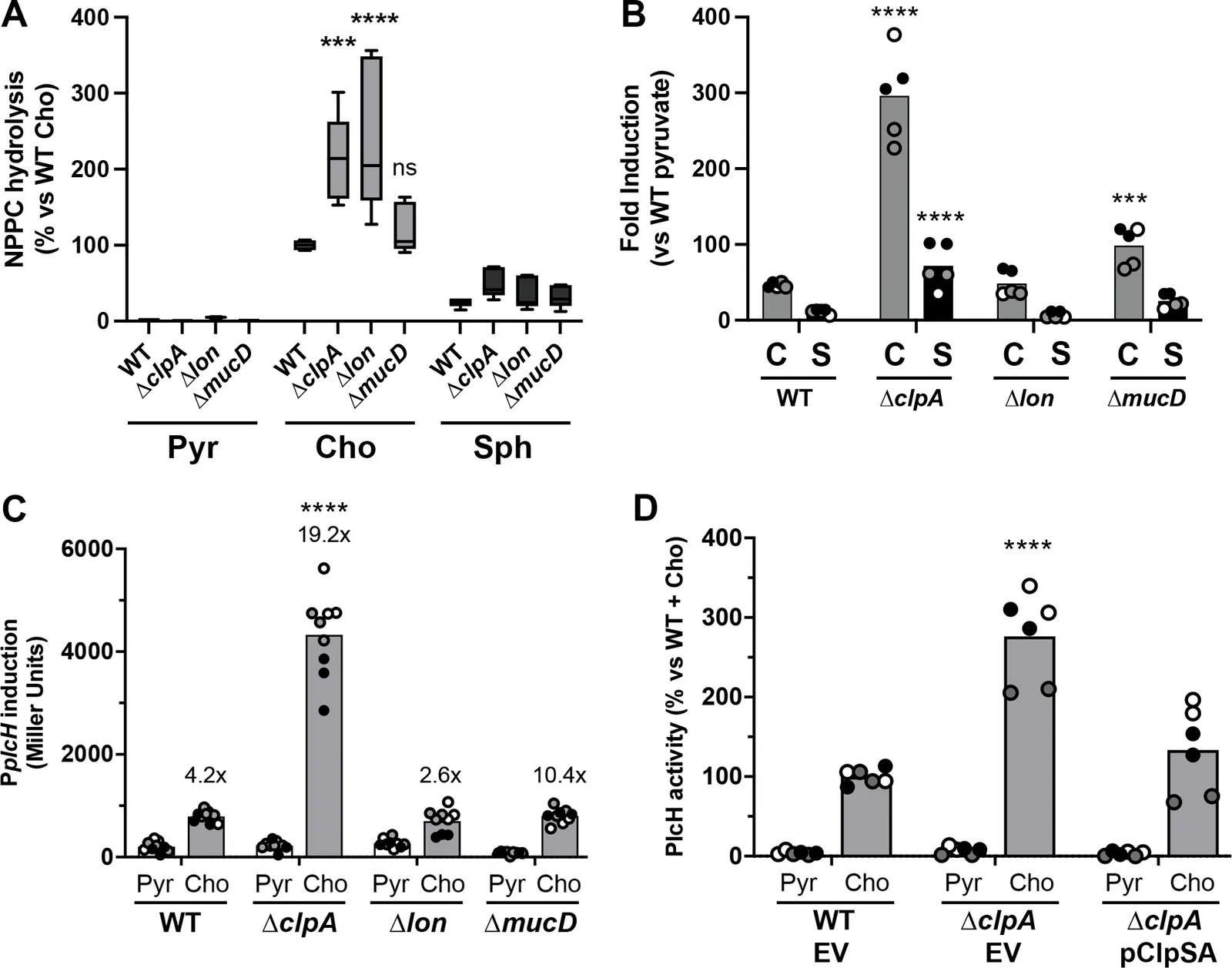
Impact of protease-related genes on PlcH activity. (**A**) PlcH activity of WT and mutants of *clpA*, *lon*, and *mucD* in uninduced (pyruvate, Pyr) compared to induction with choline (Cho), or sphingosine (Sph). NPPC hydrolysis activity normalized to WT + choline as 100%. (**B**) Fold induction of NPPC hydrolysis activity based on the activity data presented in panel **A**. Abbreviations: C, choline; S, sphingosine. (**C**) Transcriptional reporter assay showing expression from the pMW22 P*_plcH_-lacZ* reporter plasmid comparing pyruvate and choline induction in each strain. The numbers over the bars for each strain denote the fold change between pyruvate and choline conditions for this transcriptional reporter. (**D**) Complementation of NPPC hydrolysis activity with *clpSA* expression. Statistical analysis for all by two-way ANOVA with Dunnett’s post-test within each induction condition with WT as the comparator strain (***, *P* < 0.001; ****, *P* < 0.0001). All data points are shown and are colored by experiment, with white circles for all replicates from experiment #1, gray from experiment #2, and black from experiment #3, and the overall means are represented by the bars. Only the means from each experiment are used in the statistical analyses for these panels (i.e., *n* = 3 per condition).

Among our top mutants for PlcH overproduction were mutants with transposon insertions in the *clpA*, *lon*, and *mucD* genes (Table 1), each of which encodes a protease or component of a protease complex. We generated a clean deletion for each of these genes and, while generally phenocopying transposon insertants for overproduction (Fig. 5A), we noted that for the *clpA* and *mucD* deletions, the phenotype was dependent on both the media in the plate used for recovery from the −80°C and the multi-well plate well size used during induction. Note, particularly, that ∆*mucD* shows trends in these experiments based on starting media and well size (Fig. S3), but only its fold induction in choline (Fig. 5B) is statistically significant. The *lon* deletion shows overproduction at baseline and overproduction during induction; thus the fold induction between baseline and induced is not much higher than that of WT. Conversely, ∆*clpA* and ∆*mucD* both show PlcH activity in the uninduced condition similar to or lower than WT but a larger difference (fold induction) between uninduced and choline-induced conditions (Fig. 5B) that varies based on the plate and induction conditions (data for all media and well combinations are shown in Fig. S3).

To determine whether PlcH overproduction in these strains occurred at the level of choline-dependent transcriptional induction, we used our previously described P*_plcH_-lacZ* reporter plasmid, pMW22 (28). Deletion of *clpA* led to an increase in reporter activity from the *plcH* reporter compared to WT (Fig. 5C). Deletion of *lon* and *mucD* did not increase total *plcH* reporter expression during induction with choline, but the *lon* deletion mutant showed higher promoter activity in the uninduced condition (and thus lower fold induction), while the *mucD* deletion mutant showed lower promoter activity in the uninduced condition (thus higher fold induction) (Fig. 5C).

We have had long-term interest in *plcH* transcriptional regulation and thus wanted to further verify the impact of *clpA* mutation on PlcH expression. In addition to the *clpA* transposon mutant in Table 1, there is an additional *clpA* insertant in the library (1_2:B6) that showed ~260% PlcH production (Table S1). These two independent transposon mutants phenocopy a *clpA* deletion mutant, and a plasmid containing the *clpSA* operon complemented the PlcH overproduction phenotype (Fig. 5D).

### PlcH secretion in *plcR* mutants

**Fig 6:**
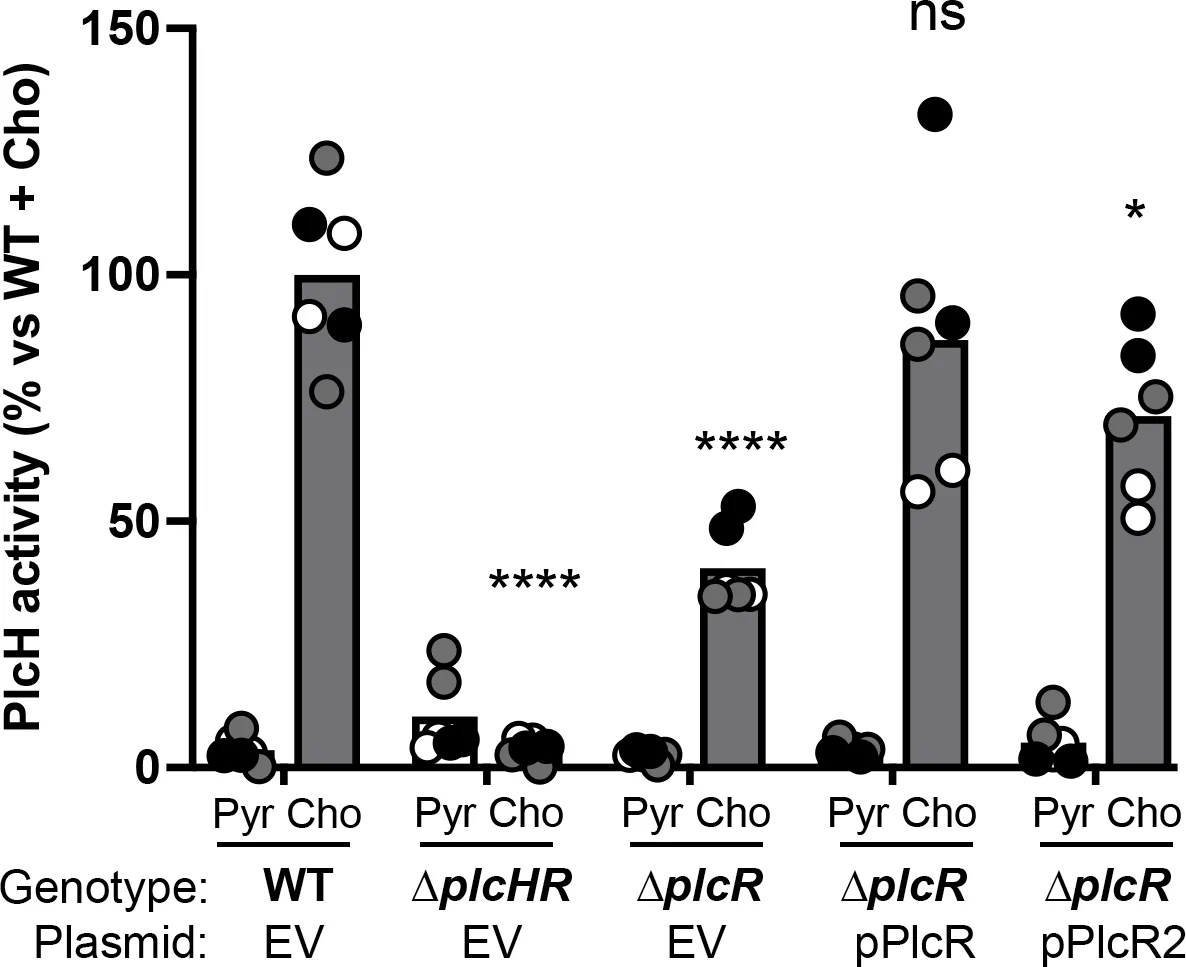
The impact of *plcR* on PlcH activity in PA14. Normalized NPPC hydrolysis activity in morpholinepropanesulfonic acid (MOPS) pyruvate (Pyr) or MOPS pyruvate and choline (Cho) from *P. aeruginosa* PA14 WT, ∆*plcHR*, and ∆*plcR* carrying the pUCP22 vector (EV), the full *plcR* gene encoding both PlcR1 and PlcR2 (pPlcR), or only the *plcR2* gene (pPlcR2). Statistical analysis by two-way ANOVA with Dunnett’s post-test within each condition, with WT as the comparator strain (****, *P* < 0.0001). All data points are shown and are colored by experiment, with white circles for all replicates from experiment #1, gray from experiment #2, and black from experiment #3, and the overall means are represented by the bars. Only the means from each experiment are used in the statistical analyses for these panels (i.e., *n* = 3 per condition).

Transposon insertants in the *plcR* gene failed to make our cutoff for Table 1, even though *plcR* deletion mutants were previously shown to have markedly reduced supernatant PlcH activity in PAO1 (14, 25). Further examination of the insertion sites for the two *plcR* transposon mutants in the PA14 library (Table S1) shows that both have insertions prior to the start of the second in-frame open reading frame, termed *plcR2*. Thus, both transposon insertions disrupt the *plcR1* product but do not insert into the *plcR2* product, and *plcR2* is sufficient for PlcH secretion (25). Since the work on *plcR* and the initial investigation into the roles of *plcR1* and *plcR2* were previously conducted only in PAO1, we generated a *plcR* deletion mutant in PA14 and tested complementation with the empty vector, *plcR (plcR1* and *plcR2),* or *plcR2* alone. In Fig. 6, we show that both the *plcR* and *plcR2* genes show a similar function in PA14 as they do in PAO1. While reports in PAO1 show about a 90% reduction in NPPC hydrolysis activity in ∆*plcR* supernatants compared to WT, here we show that the PA14 ∆*plcR* strain has about a 60% reduction in NPPC hydrolysis activity in the supernatant. These differences may be partly due to media and induction time differences between the studies but could certainly be strain-dependent differences in the importance of PlcR in PlcH folding, secretion, and/or activity.

### The impact of mutations in the choline catabolic pathway on PlcH production

**Fig 7:**
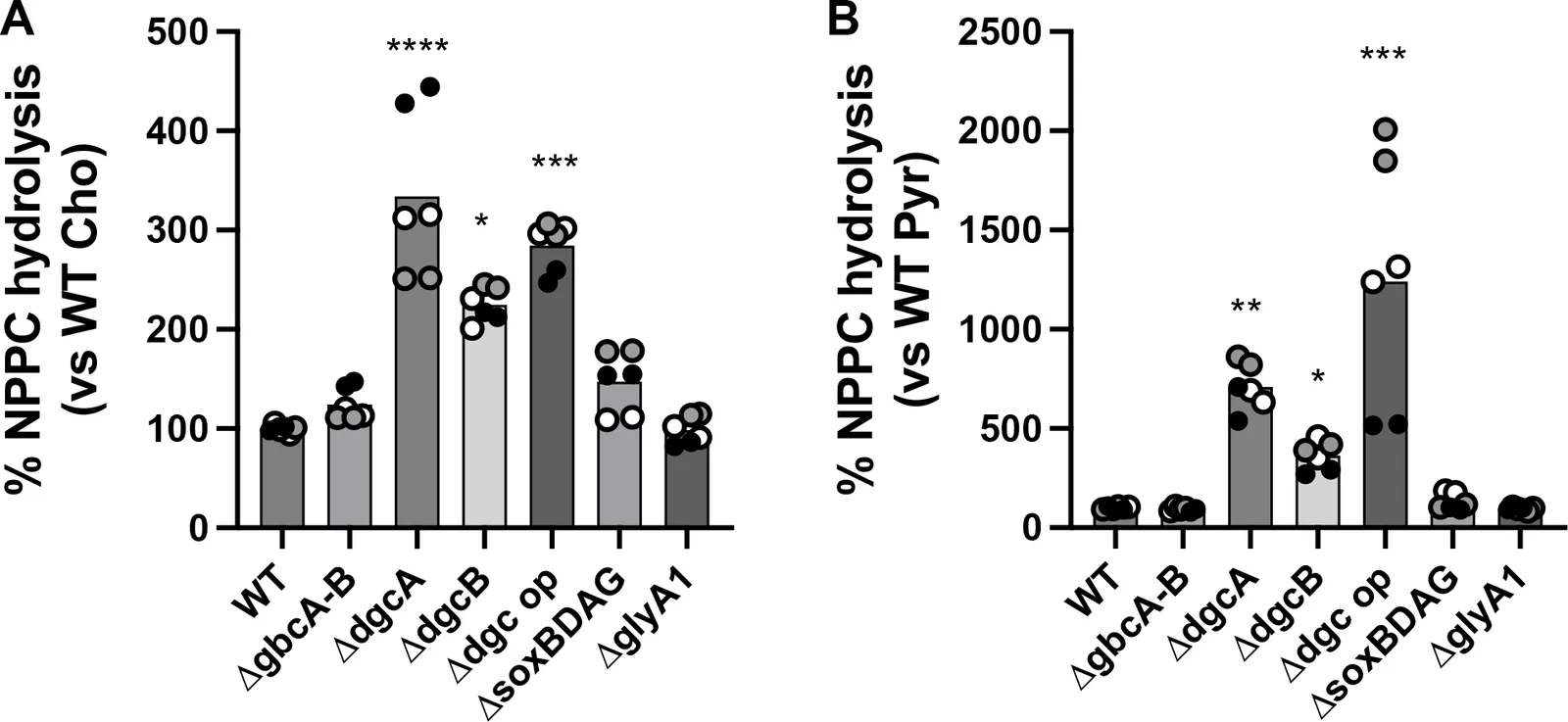
PlcH activity is altered for some mutants in the choline catabolic pathway. NPPC hydrolysis activity measured (**A**) under choline induction conditions or (**B**) at baseline pyruvate conditions. Statistical analysis for each panel by ANOVA followed by Dunnett’s post-test with WT as the comparator strain (*, *P* < 0.05; **, *P* < 0.01; ***, *P* < 0.001; ****, *P* < 0.0001). All data points are shown and are colored by experiment, with white circles for all replicates from experiment #1, gray from experiment #2, and black from experiment #3, and the overall means are represented by the bars. Only the means from each experiment are used in the statistical analyses for these panels (i.e., *n* = 3 per condition). The “∆dgc op” strain is a deletion of the *dgc* operon: ∆*PA14_71240-PA14_71250-dgcA-dgcB*.

Two transposon insertants with both increased baseline and choline-induced PlcH activity were in the *dgcB* and *glyA1* genes that are part of the choline catabolic pathway (45). The impact of these mutations during choline-dependent induction was anticipated (46), but the increase in PlcH baseline production was unexpected. To follow up on this observation, we measured PlcH production in uninduced and choline-induced conditions, comparing WT to deletion mutants in genes for each step of the *P. aeruginosa* choline catabolic pathway (Fig. 7A and B). In the choline-induced condition, deletion of *dgcA*, *dgcB*, or the entire *dgc* operon (*PA5396*, *PA5397*, *dgcA*, and *dgcB*) resulted in increased NPPC hydrolysis activity (Fig. 7A). In the baseline condition, these three mutants were also the only ones to show a significant increase in activity (Fig. 7B), though there was much more variability in the basal phenotype than the induced phenotype, and deletion of *dgcA* had a greater impact than deletion of *dgcB*. The reason for increased basal activity in mutants of the *dgc* locus is not known, though we speculate in the Discussion.

Interestingly, while the *glyA1* transposon insertion mutant showed increased PlcH activity at baseline (Table 2), we did not see that in either the ∆*glyA1* or the ∆*soxBDAG* strains (Fig. 7B). The *glyA1* gene is the first gene in the *glyA1soxBDAG* operon, so perhaps a deletion of the whole operon would show high baseline PlcH activity, mimicking the *glyA1* transposon mutant, though we have not tested that hypothesis.

### Further examination of two additional genes identified in this screen

**Fig 8:**
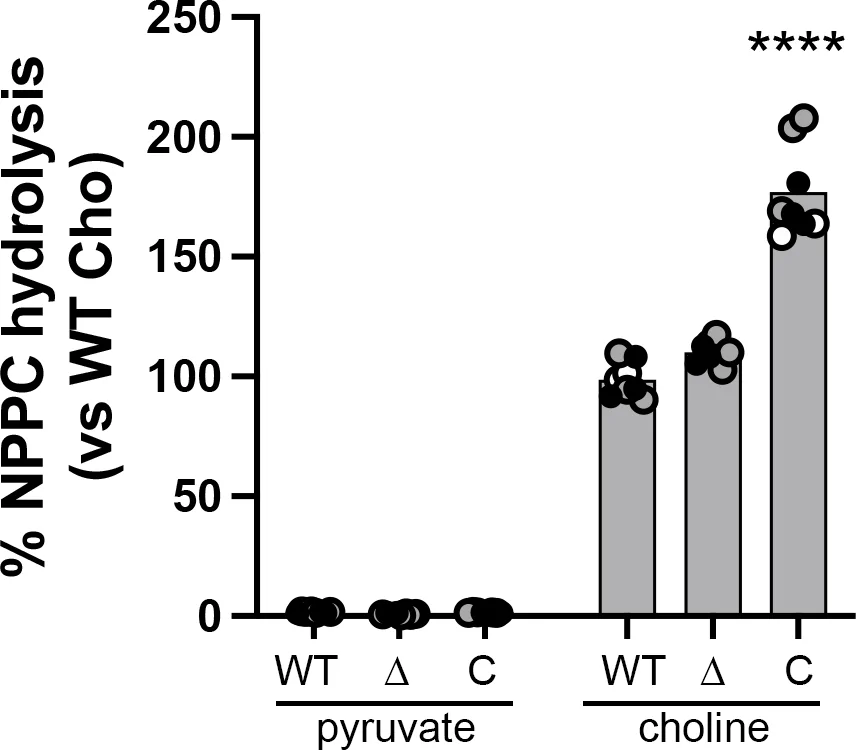
Overexpression of *rmcA* increases PlcH expression. PlcH activity, as measured by NPPC hydrolysis, comparing WT, ∆*rmcA* (∆), and the ∆*rmcA attTn7::rmcA* complemented strain (**C**) in pyruvate and choline. Statistical analysis by two-way ANOVA with Dunnett’s post-test within each condition, with WT as the comparator (****, *P* < 0.0001). All data points are shown and are colored by experiment, with white circles for all replicates from experiment #1, gray from experiment #2, and black from experiment #3, and the overall means are represented by the bars. Only the means from each experiment are used in the statistical analyses for these panels (i.e., *n* = 3 per condition).

Transposon insertion into *rmcA* led to both increased baseline and choline-induced PlcH production (Tables 1 and 2; Table S1). The *rmcA* gene is a multifunctional protein containing the cyclic-di-GMP-related domains GGDEF and EAL as well as a redox-related PAS domain and is involved in *P. aeruginosa* biofilm formation (47). We received the ∆*rmcA*, WT parent, and *attTn7* complementation strains from George O’Toole (Geisel School of Medicine at Dartmouth) and measured PlcH production at baseline and during induction with choline (Fig. 8). The ∆*rmcA* strain shows neither increased baseline nor increased choline-induced NPPC hydrolysis activity, but the complemented strain does show a significantly higher level of PlcH activity during choline induction. To better understand the discrepancy between the transposon mutant observation and the clean deletion, we examined the position of the transposon insertion. The *rmcA* transposon insertant that made our PlcH hyperproduction list was 8_3:B8, with the transposon inserting at base 335 of 3738. There is a second transposon insertant in the PA14 library, 15_3:F11 (insertion at base 3425 of 3738), that has no significant PlcH expression phenotype. The 15_3:F11 insertion cuts off the C-terminal EAL domain, whereas the 8_3:B8 insertion falls in the periplasmic domain. The positioning of the transposon insertion changes the phenotype of the transposon mutant strains, and while the mechanism is not known, it is tempting to speculate that expression of a C-terminal portion of RmcA in the 8_3:B8 insertant might yield this phenotype.

**Fig 9:**
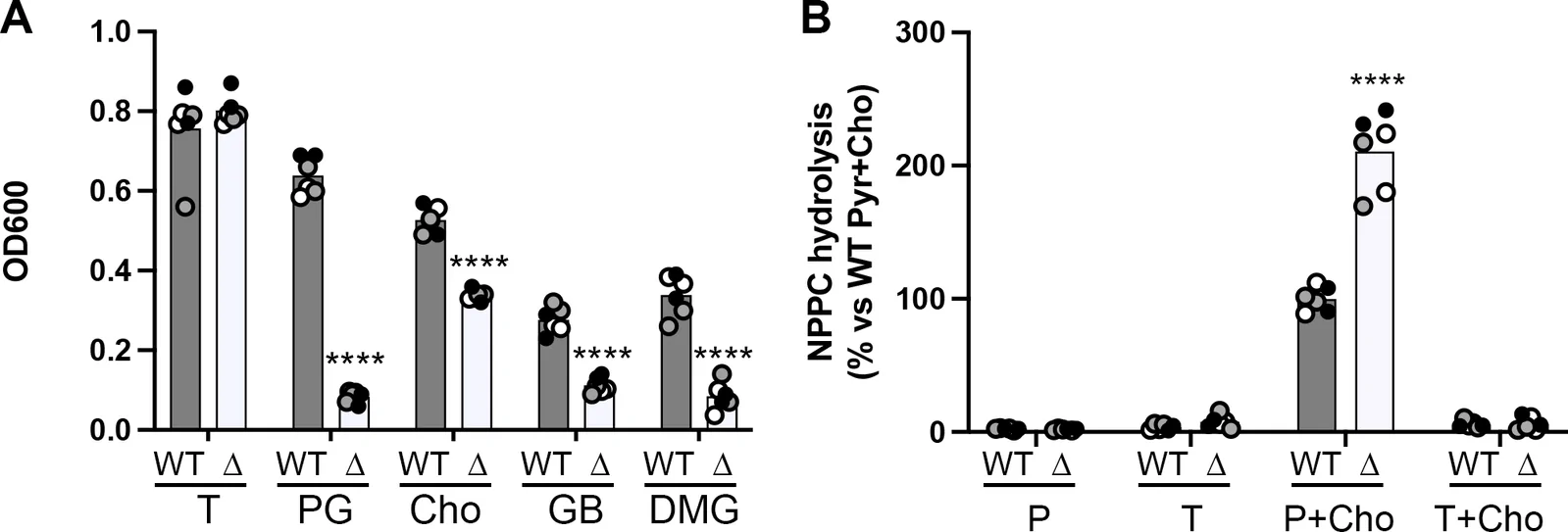
Deletion of *ridA* (*PA5339*) leads to increased PlcH expression correlated with growth defects on choline and its metabolites. (**A**) Growth of WT vs ∆*ridA* (∆) at 24 h, as measured by OD_600_ from cells started at OD_600_ = 0.05. MOPS media with no carbon source was the base, and the added carbon sources are noted: 0.05% tryptone broth (T), 20 mM pyruvate + 5 mM glucose (PG), 20 mM choline (Cho), 20 mM GB, or 20 mM DMG. (**B**) PlcH activity measured as NPPC hydrolysis from cells grown as overnights in MOPS + 0.05% tryptone broth and moved to the listed conditions for induction. MOPS media with no carbon was the base media, and the added compounds are noted: 20 mM pyruvate (P), 0.05% tryptone broth (T), 20 mM pyruvate + 2 mM choline (P + Cho), or 0.05% tryptone broth + 2 mM choline (T + Cho). Statistical analysis for both panels done with two-way ANOVA with Sidak’s post-test, comparing WT to the *ridA* deletion strain in each condition (****, *P* < 0.0001). All data points are shown and are colored by experiment, with white circles for all replicates from experiment #1, gray from experiment #2, and black from experiment #3, and the overall means are represented by the bars. Only the means from each experiment are used in the statistical analyses for these panels (i.e., *n* = 3 per condition).

*PA5339* encodes RidA, loss of which impacts growth and motility via accumulation of 2-aminoacrylate (48). In our initial screen, the *PA5339::Tn* strain had a low PlcH activity phenotype (Table 1). We deleted the *ridA* gene and noticed immediately that it did not grow well in our standard overnight culture conditions (MOPS pyruvate + glucose). The *ridA* deletion also had growth phenotypes on choline, glycine betaine, and dimethylglycine, but not in MOPS with 0.05% tryptone (Fig. 9A). When we grew overnight cultures in the MOPS + 0.05% tryptone and then transferred to standard induction conditions, we noted increased NPPC hydrolysis in the ∆*ridA* mutant in the MOPS pyruvate + choline condition compared to WT (Fig. 9B). This suggests that the initial screen phenotype was likely impacted by limited cell growth, but that changing the pre-growth conditions uncovered overproduction of PlcH in the *ridA* mutant based on its inability to metabolize choline.

## DISCUSSION

PlcH is an important virulence factor in *P. aeruginosa,* and while focused investigations have uncovered more about its function and regulation recently, here we chose to explore PlcH regulation by screening the entire PA14 transposon mutant library for PlcH enzymatic activity. In doing so, we identified a number of genes in which insertion alters PlcH activity—some known, some suspected, and some unknown. We chose to follow up on a subset of these genes and present new connections between these pathways and PlcH.

For the most part, the impact of mutations in the choline metabolic pathway on PlcH activity was predicted based on previous results. What was not predicted was the much higher baseline activity of mutants in the genes of the *dgc* operon (*PA5396-PA5397-dgcA-dgcB*). In our examination of the GbdR regulon (46), we only compared the *dgcA* mutant to WT in the choline condition, where hyperinduction of *plcH* and other GbdR-regulated transcripts was noted. Here, we observed the high basal levels of PlcH activity in these mutants (Fig. 7). This suggests two possibilities. First, even though the strains are picked into media with no choline from the plate and grown overnight, it is possible that the population or some proportion of cells in the population retains enough choline from the plates to produce the observed induction. The second possibility is that there is synthesis of dimethylglycine or some related metabolite sensed by GbdR at a very low baseline level in *P. aeruginosa* that is normally removed by action of the Dgc enzymes but builds up in their absence. We continue to examine both possibilities.

In addition to the known and suspected pathways identified, we were surprised by the phenotypes for *clpA*, *mucD*, and *lon* mutants (Fig. 5). The mutants in these genes encoding proteases (MucD and Lon) or the ClpX protease-associated chaperone (ClpA) showed increased PlcH activity, which, for *mucD* and *clpA* mutants, was dependent upon growth conditions (Fig. S3). The function of Lon appears to be the most straightforward. Loss of *lon* results in increased baseline and induced PlcH activity (Fig. 5A), and while there seems to be a small impact on transcription in the lon mutant (Fig. 5C), we suspect that PlcH is likely a substrate for Lon. Conversely, loss of *clpA* results in greater induction of both PlcH enzyme activity (Fig. 5A and B) and transcriptional induction of *plcH* (Fig. 5C). Though tempting to implicate regulation through the choline metabolic pathway, the *clpA* mutant also shows much higher PlcH activity than WT in the presence of sphingosine (Fig. 5B), suggesting a broader impact of ClpA on *plcH* transcription. To date, our data do not resolve how much of the *mucD* phenotype is dependent on alteration of *plcH* transcription versus MucD directly targeting PlcH for degradation in the periplasm. It is apparent from the phenotypes of these protease mutants that stability of PlcH is something that needs to be examined in future work.

The *clpA* gene is retained in all Pseudomonads, but, surprisingly, is frequently mutated during long-term *P. aeruginosa* infection in the lungs of people with cystic fibrosis (49–52). During *in vitro* evolution for growth with ceftazidime, *clpA* mutants are selected in both standard laboratory strains and hypermutator lineages (53–55), and *clpA* mutants also arise *in vitro* during growth in aztreonam (52). These *in vivo* and *in vitro* mutations are predominantly in conserved residues or result in frameshifts, suggesting that many of these alleles are loss of function. Experimentally, transposon insertions in *clpA* result in lower virulence in *Caenorhabditis elegans* (56), but, conversely, increased survival in the murine lung (57). These findings all point to ClpA playing an important and probably multifaceted role in the CF lung and in response to ceftazidime and aztreonam. Here, we demonstrate that *clpA* deletion increases *plcH* transcription and resultant secreted PlcH activity. It remains to be tested whether the *clpA* mutants arising in CF and in these *in vitro* selections always result in PlcH overexpression.

One of the weaknesses of this screen is that its cost- and time-intensive enzymatic assay meant that the whole library could only be screened once. Thus, there are certainly false positives and false negatives present in these data that replication could have eliminated. As a discovery process, we were not particularly worried about false negatives, especially given the number of mutants with interesting phenotypes. Since the library was screened for choline responsiveness or altered baseline levels of PlcH, we missed *sphR* and any genes related to induction independent of choline. Also, the library does not contain mutants in all non-essential genes; for instance, the ncRNA *phrS* is nonessential and known to impact PlcH (37), but does not have a transposon mutant in the library. Based on the genes previously known to have altered PlcH activity that had transposon insertions in the library, we hit 23 of 24, though not all were robust enough to be included in Table 1, suggesting a rough estimate of 4% for a false negative rate. False positives are more problematic in such a screen, and it is important to assess false positives that were methodological or stochastic versus false positives based on the nature of the transposon insertion site. Of the transposon mutants we screened secondarily (Fig. 4) or tested with clean deletions, we had no transposon mutants whose phenotypes failed to repeat. We did, however, have a number of transposon mutant phenotypes that were not matched by the phenotype of the clean deletion, but in which the gene had some role in PlcH activity (*rmcA*, *cerN*, *ridA*), and one with no phenotype at all (*PA5533*). In addition, we tried to make a deletion mutant of *PA2797* multiple times, but of dozens of double-crossover colonies screened from three independent single crossovers, all were WT revertants.

Our work presented here and elsewhere, as well as all studies of PlcH that we know of, analyze PlcH regulation in liquid media. However, a few lines of evidence point to a role in c-di-GMP and surface sensing. First, RmcA can regulate c-di-GMP production and biofilm formation (47), and there is a PlcH overproduction phenotype in the *rmcA::Tn* and overexpressor (Tables 1 and 2, and Fig. 8). Second, the impact of culture well volume on the ∆*mucD* and ∆*clpA* strains (Fig. 5; Fig. S3) is reminiscent of impacts of surface-to-volume ratios and potentially attachment. Third, based on the impact of the *orn::Tn* mutant (Table 1), where mutants in *orn* are known to have high intracellular c-di-GMP due to decreased degradation (58, 59). Altogether, these suggest the potential for a previously unappreciated contribution of c-di-GMP and surface sensing in PlcH regulation that will be a focus of future work.

We were not the first group to screen for mutants in PlcH activity. In 1997, Mike Vasil and colleagues reported results from a screen of ~7,500 random Tn5 transposon mutants for altered PlcH enzyme activity (30). They had two hits that passed the secondary screen: *betB*, which encodes the betaine aldehyde dehydrogenase, and *truB* (*PA4742*, *PA14_62730*), which encodes the tRNA pseudouridine 55 synthase. Transposon mutants for both of these are present in the PA14 library, and we covered *betB* along with other genes involved in choline metabolism in the Results section. In our screen, the *truB* insertant showed ~55% of normalized NPPC hydrolysis activity. In addition to *truB,* a few other tRNA modification-related genes had alterations in PlcH activity in our screen to a similar scale as *truB*, including *PA4852*, encoding a putative tRNA dihydrouridine synthase, and *PA3626*, encoding a putative tRNA pseudouridine synthase D. Conversely, transposon insertion into *trmA* (*PA4720*), encoding the tRNA uracil 5-methyltransferase, and *trmD*, encoding a tRNA guanine-N1 methyltransferase, both showed modest increases in PlcH activity. Interestingly, while *trmD* is essential in *P. aeruginosa*, the transposon mutant in the library has the insertion at base 757 of 759. Vasil and colleagues previously noted that a *truB* mutant had more abundant *plcH* transcript but very low PlcH enzyme activity (30). Along with *truB*, the phenotypes of mutants in tRNA modification genes suggest there may be some translational regulation of PlcH expression that remains unexplored.

As with any genome-wide screen, there remains a lot of interesting genes that we have not followed up with additional experiments. One area of interest is genes related to zinc, including the >300% PlcH activity levels of mutants in the zinc-related genes *zur* (*np20*) and *dksA2* (Table 1; Fig. 4). DksA2 is an RNA polymerase-binding transcription factor that is homologous to DksA except that it is induced during zinc starvation and does not require zinc binding for its function (60). Zur is a zinc-binding transcription regulator that represses expression of zinc uptake and scavenging systems when bound to zinc (61). In addition to these large effects, insertion into the periplasmic binding protein of the Znu ABC transporter, *PA5498*, resulted in 150% PlcH activity, while insertion into *znuC* (*PA5500*) resulted in 120% PlcH activity. These point to a role for zinc in PlcH regulation. At this time, we do not know if the signal of zinc starvation works directly on PlcH at some level or is mediated by affecting choline metabolism, cofactors of which require zinc, but something we are interested in exploring.

Our findings in this screen and its follow-up experiments cement the role of known and suspected genes, providing more detail on their impact and verifying function in the *P. aeruginosa* strain PA14. Beyond those, the screen also identified a number of genes that impact PlcH activity that were unexpected. Some of these were characterized here, but the larger proportion remains unexplored. Likely, others will be able to discover connections between their genes of interest and the production of this important *P. aeruginosa* virulence factor using the data presented here.

## MATERIALS AND METHODS

### Strains and growth conditions

Strains were stored as glycerol stocks at −70 or −80°C and recovered on Pseudomonas Isolation Agar (PIA) or PIA with 50 µg/mL gentamicin as appropriate for the strain. Specific instances of recovery on LB from freezer stocks are noted in the Results section. During genetic manipulations, *P. aeruginosa* was selected for, and *Escherichia coli* eliminated, using PIA plates supplemented with 50 µg/mL gentamicin. Prior to PlcH induction studies, *P. aeruginosa* was grown overnight in MOPS medium (62), modified as previously described (63), and supplemented with 20 mM pyruvate, 5 mM glucose, and 25 µg/mL gentamicin when appropriate.

### Generation of deletion mutants and complementation constructs

All PCRs were conducted using Q5 DNA polymerase (NEB). Primer sequences are listed in Table S2. All plasmids created for this study were sequenced using Plasmidsaurus (Eugene, Oregon, USA). For plasmid carriage, *P. aeruginosa* and *E. coli* strains were transformed via electroporation and selected for growth on PIA with 50 µg/mL gentamicin and LB with 10 µg/mL gentamicin, respectively.

The allelic exchange vectors for the deletions generated in this study in Table 3 were built using HiFi assembly (NEB) between a synthetic DNA block (IDT or Twist) and HindIII- and KpnI-digested pMQ30. After transformation into DH5α, miniprep, and sequencing, clones with correct and identical sequences were pooled for transformation into the conjugative donor *E. coli* strain S17λpir. Plasmid sequences for each of the deletion constructs are available in File S1. Donor *E. coli* were mixed with their appropriate recipient strain, and conjugation was allowed to occur in spots on LB plates at 30°C overnight. Conjugation spots were resuspended and plated on PIA with 50 µg/mL gentamicin to select for single-crossover integrants. After restreaking onto PIA with 50 µg/mL gentamicin, single-crossover integrants were moved to LB without selection for 3 h prior to plating on LB with 5% sucrose and without salt at 30°C overnight. Sucrose-resistant colonies were screened for loss of gentamicin resistance prior to PCR screening to determine whether each double-crossover colony was a mutant or WT revertant. PCR primer sequences for screening of each deletion mutant are listed in Table S2.

**TABLE 3:** Strains and plasmids used in this study

Strains		
Database #	Genotype	Source
MJ1164	PA14 WT	(12)
MJ1201	∆*plcHR* (restock of MJ144)	(29)
MJ1225	∆*plcR*	This study
MJ1059	∆*tatABC*	This study
MJ1272	∆*xcpRSTUVMXY*	This study
MJ1220	∆*lon*	This study
MJ1222	∆*mucD*	This study
MJ1138	∆*clpA*	This study
MJ587	∆cerN	(64)
PD146	PA14 WT *attTn7*::empty	This study
PD148	∆*cerN attTn7*::empty	This study
PD150	∆*cerN attTn7*::*cerN*	This study
GO232	PA14 WT	(47)
GO6279	∆rmcA	(47)
GO8813	∆*rmcA attTn7*::PBAD-*rmcA*	(47)
MJ288	∆*gbcA-B*	(45)
MJ336	∆*dgcA*	(45)
MJ1211	∆*dgcB*	This study
MJ1214	∆*dgc* operon (*PA5396-dgcB*)	This study
MJ1302	∆glyA1	This study
MJ1305	∆*soxBDAG*	This study
MJ1237	∆*ridA* (*PA5339*)	This study
MJ1295	∆*PA5533*	This study

Plasmids		
Database #	Desc	Source
pUCP22	Empty expression vector	(65)
pMQ30	Allelic exchange vector	(66)
pTNS3	Tn7 transposase expression	(67)
pFLP2	Flp recombinase expression	(68)
pMW22	P*plcH-lacZYA* reporter plasmid	(28)
pMW247	pPlcR2 complementation vector	This study
pMW258	pPlcR complementation vector	This study
pMW266	pClpSA complementation vector	This study
pPD42	*cerN* complementation via *attTn7*	This study

For all but the *cerN attTn7* complementation vector (pPD42), complementation constructs were generated by HiFi assembly of synthetic DNA fragments (Twist) into HindIII- and KpnI-cut pUCP22, putting each construct under the control of its native promoter in opposite orientation to the transcription of the *lacZ* alpha fragment. Plasmid sequences for each complementation vector are available at File S1.

The plasmid enabling chromosomal *attTn7::cerN* integration was generated by amplification from PA14 genomic DNA with primers #2869 and #2870 to generate a 2,420 bp PCR fragment that was digested with HindIII and ligated into HindIII-cut pUC18-mini-Tn7T (69). After transformation into *E. coli* and miniprep, orientation was assessed by BamHI digest, and clones with proper orientation were sequenced. The pPD42 sequence is available in File S1. To generate the chromosomal complementation and control strains, the ∆*cerN* mutant and PA14 WT were co-electrotransformed with either pPD42 or pUC18T-miniTn7T-Gm along with pTNS3 based on the methods of Schweizer and colleagues (69). Integrants at the *attTn7* locus were selected by growth on gentamicin, and insertion was verified by PCR. The gentamicin resistance cassette was excised by FLP-mediated recombination by electrotransforming the target strains with pFLP2, selecting on carbenicillin, and subsequently screening for loss of gentamicin resistance (69). Loss of the gentamicin resistance gene was verified by PCR with primers #2771 and #2772 (Table S2).

### Screen of the PA14 transposon mutant library for NPPC hydrolysis activity

To screen the PA14 transposon mutant library (38), between two and four library plates per day were replicated onto LB plates with 10 µg/mL gentamicin using a multipin device and incubated overnight at 37°C. These overnight plates were replicated into 96-well polystyrene plates containing 200 µL/well of MOPS media with 20 mM pyruvate, 5 mM glucose, and 5 µg/mL gentamicin using a multipin device. The 96-well plates were sealed with a sterile, gas-permeable membrane (Microporous Sealing Film, USA Scientific) and incubated with horizontal shaking at 37°C for 18 h. Auxotrophs were defined as having an increase in OD_600_ of less than 0.05 in the overnight growth plate.

From each column of the overnight plates, 50 µL was moved to two separate columns of a new plate with 150 µL of MOPS media, one column with 20 mM pyruvate and the other column with 20 mM pyruvate + 2 mM choline. Thus, each well from the overnight growth 96-well dish was moved to a non-inducing condition well (no choline) and an inducing condition well (2 mM choline), with each initial overnight plate now distributed over two 96-well plates. These induction plates were sealed with a gas-permeable membrane and incubated at 37°C with horizontal shaking for 3 h. Multichannel pipetting was used to move 75 µL from each well into a fresh 96-well plate. After all wells were transferred, 75 µL of 2× NPPC reaction buffer (based upon the Kurioka and Matsuda method [70], modified as we have described previously [28], but using a final concentration of 10 mM NPPC) was added with mixing by gentle pipetting, and plates were moved immediately to a pre-warmed (37°C) plate reader where, after the initial OD_600_ reading, plates were read at 410 nm every 5 min for 30 min. To determine the percent induction for NPPC hydrolysis activity, the change in 410 nm absorbance over time in the baseline (uninduced) condition was subtracted from the change in 410 nm absorbance over time in the induced (2 mM choline) condition. This delta-NPPC hydrolysis number was divided by the average delta-NPPC for that plate’s induced wells, not including auxotrophs and the well itself. Thus, percent NPPC hydrolysis is within-plate and within-day normalized for each well. The resulting log2 normal distribution (Fig. 2) supports that this was a reasonable normalization strategy.

To determine the percent baseline NPPC hydrolysis, the change in 410 nm absorbance over time in the baseline (uninduced) condition was calculated and divided by the average delta-NPPC for that plate’s uninduced wells, not including auxotrophs and the well itself. Since the NPPC hydrolysis level is very low in WT and most of the transposon mutants, we focused only on mutants in which NPPC hydrolysis activity was increased.

We did not re-verify transposon insertion site except in two cases: (i) where the clean deletion mutant failed to recapitulate the phenotype of the transposon insertion, (ii) where we were unable to make a clear deletion. The insertion site verifications in these cases were done by rescue cloning of the transposon. Briefly, genomic DNA from the respective transposon mutant was digested with KpnI and ligated into KpnI-cut, SAP-treated pUC19. After transformation into DH5α and selection on gentamicin, plasmids were prepared by miniprep and sequenced to identify the insertion sites.

### Induction and measurement of PlcH activity following the initial screen

For standard PlcH enzymatic assays, *P. aeruginosa* strains were grown overnight in MOPS minimal media with 20 mM sodium pyruvate, 5 mM glucose, with 20 µg/mL gentamicin if appropriate. Cells were collected via centrifugation, washed in MOPS minimal media, and resuspended in MOPS media with 20 mM pyruvate or in MOPS with 20 mM pyruvate plus 2 mM choline or 50 µM sphingosine (Cayman Chemicals). All inductions took place at 37°C with horizontal shaking at 170 rpm for 3 h, with plates covered by breathable films.

Phospholipase C activity was measured by observing the hydrolysis of the synthetic substrate ρ-nitrophenyl-phosphorylcholine (NPPC) based upon the Kurioka and Matsuda method (70), modified as we have described previously (28), but using a final concentration of 10 mM NPPC. NPPC hydrolysis was measured by monitoring the absorbance at 410 nm over time. Phospholipase C activity was calculated as micromoles of ρ-nitrophenol generated per minute of reaction per optical density (OD_600_). ρ-Nitrophenol concentration was calculated using the extinction coefficient of 17,700 M^−1^ cm^−1^ (71). Most of the PlcH activity data presented herein are normalized to the intraday activity of WT in either baseline or induced conditions, as noted in the Results section and figure legends.

### Assessment of transcription induction using a *plcH-lacZ* reporter

To assess changes to transcription from the *plcH* promoter, we conducted β-galactosidase assays using a plasmid-based P*_plcH_-lacZ* transcriptional fusion as previously described (28), using Miller’s method (72). Induction conditions were identical to those described above for PlcH enzyme activity. Data were reported as Miller units, and fold induction was calculated relative to each strain’s uninduced (pyruvate) condition.

### Graphing and statistical analyses

All graphs were generated in GraphPad Prism after normalization and other relevant calculations in Microsoft Excel. Statistical analyses were based on one-way or two-way ANOVA with appropriate post-tests. We focused on differences in mutants compared to WT in nearly all experiments, as we know the conditions we use for induction are different from baseline and different from each other from previous work.

## SUPPLEMENTAL MATERIAL

File S1 jb.00231-26-s0001.txt Sequences for each construct used in the study.

Supplemental figures jb.00231-26-s0002.pdf Figures S1 to S3.

Table S1 jb.00231-26-s0003.xlsx Complete NPPC hydrolysis results for the PA14 transposon library.

Table S2 jb.00231-26-s0004.xlsx All primers used in this study.

ASM does not own the copyrights to Supplemental Material that may be linked to, or accessed through, an article. The authors have granted ASM a non-exclusive, world-wide license to publish the Supplemental Material files. Please contact the corresponding author directly for reuse.
